# Brugada syndrome: a review and the role of epicardial ablation in management

**DOI:** 10.1186/s43044-024-00485-3

**Published:** 2024-05-07

**Authors:** Osama Jema Abuzuagaia, Khalid Abozguia, Yousef Darrat

**Affiliations:** 1 University of Misurata, Radio Street, Misurata, Libya; 2 Marshall University, Huntington, WV USA; 3 St. Joseph Hospital, Lexington, KY USA

**Keywords:** Brugada syndrome, Implantable cardioverter–defibrillator, Sudden cardiac death, Ventricular fibrillation

## Abstract

**Background:**

Brugada syndrome (BrS) is an inherited arrhythmogenic syndrome characterized by cove-shaped ST-segment elevation in leads V1–V3 and incomplete or complete right bundle branch block. BrS exhibits autosomal dominant inheritance with incomplete penetrance and a male predominance. It carries a significant risk of sudden cardiac death due to ventricular fibrillation (VF).

**Main body:**

Recent studies have highlighted the presence of epicardial fibrosis as a proarrhythmic substrate in BrS, revolutionizing our understanding of the disease's pathophysiology. Catheter ablation has emerged as a crucial intervention for symptomatic BrS patients experiencing recurrent episodes of ventricular tachycardia (VT) or VF. By potentially obviating the need for implantable cardioverter–defibrillator (ICD) implantation, epicardial ablation offers a promising therapeutic approach.

**Conclusion:**

This review emphasizes the significance of current evidence and ongoing research in shaping the role of epicardial ablation as a curative strategy in BrS management, highlighting its potential benefits and the necessity for further investigation.

## Background

Inherited arrhythmia syndromes contribute significantly to sudden cardiac death, presenting a challenge in their diagnosis, management, and risk stratification. Among these syndromes, Brugada syndrome (BrS) stands out as a condition that is not yet well defined in terms of its diagnosis and risk assessment for sudden cardiac death [1]. Despite advancements in understanding cardiac channelopathies, there is a need to further explore current knowledge in BrS and investigate the potential role of epicardial ablation as a therapeutic option. Future studies are anticipated to provide additional insights and more conclusive answers to address this ongoing issue.

## Main text

Artificial intelligence (AI)-assisted technologies were not used in the production this article. Authors independently searched PubMed, web of science, google scholar, Cochrane database to identify potential studies, and papers about BrS from January 1992 to February 2023. Keywords and medical subject heading search terms “Brugada syndrome,” ”sudden cardiac death,” and “epicardial ablation” were used in several combinations.

### Epidemiology of Brugada syndrome

Brugada syndrome (BrS) exhibits a relatively low incidence, estimated to be less than 1% of the population. However, despite its infrequency, this syndrome accounts for more than 10% of all cases of sudden death and can be responsible for up to 20% of sudden deaths in individuals with structurally normal hearts [2]. Notably, BrS demonstrates a clear male predominance, with an approximate male-to-female ratio of 8:1. The typical age of presentation for BrS is around 40 years, although it is important to recognize that it can manifest at a younger age too.

### Diagnosis

**Fig. 1:**
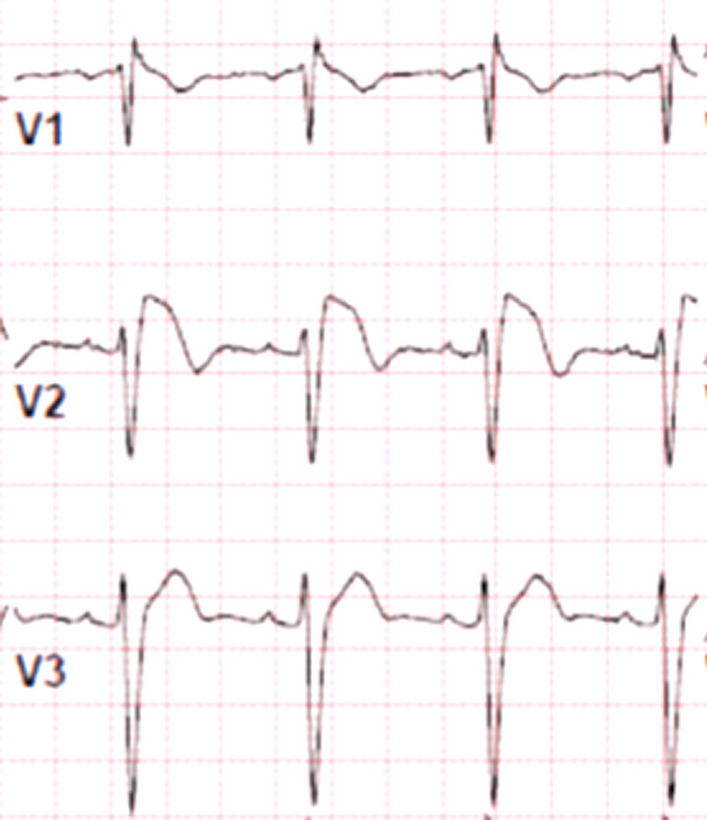
12 lead ECG showing a spontaneous Brugada pattern in leads V1 and V2

The diagnosis of BrS involves the identification of specific clinical and electrocardiographic features in patients with a structurally normal heart. It is characterized by an arrhythmogenic condition presenting with an incomplete or complete right bundle branch block pattern on the electrocardiogram (ECG) (Fig. 1). BrS often exhibits autosomal dominant familial transmission; however, there is frequently incomplete penetrance observed within affected families.

**Fig. 2:**
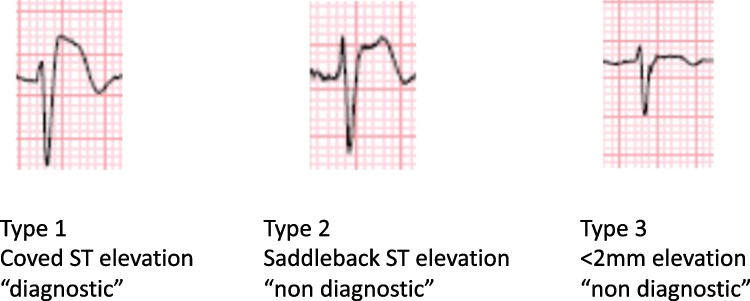
The three types of Brugada pattern ECG

A definitive diagnosis of BrS can be established in the presence of a type I spontaneous ECG pattern, which is characterized by a J-point elevation of ≥ 2 mm (Fig. 2). Clinical symptoms such as sustained ventricular arrhythmia, aborted cardiac arrest, or presumably arrhythmic syncope further support the diagnosis. In certain cases where the ECG findings are borderline, the administration of a sodium channel-blocking agent, such as flecainide or ajmaline, can "unmask" the characteristic pattern associated with BrS, aiding in the diagnosis. Additionally, recording ECG leads V1 and V2 in the second and third intercostal spaces can provide valuable diagnostic information [3] (Tables 1 and 2).

**Table 1:** Comparison between endocardial and epicardial ablation

	Endocardial approach	Epicardial approach
Ablation target	Trigger ablation Substrate modification	Substrate modification (low voltage < 1mV late potential > 80 ms after end of QRS)
Location	Septal and anterolateral RVOT	Anterior epicardial RVOT
Endpoint	Non-inducibility of VT/VF Elimination of PVC inducing VT/VF	Non-inducibility of VT/VF ECG normalization Elimination of substrate
Success rate	88% success rate (24)	96% with repeat ablation procedure (25)

**Table 2:** Epicardial ablation studies in patients with Brugada syndrome

Study	Type of study	# of patients	Preexisting ICD	Findings
Nademanee et al. (17)	Case series	9	All have preexisting ICD	No recurrence of VT or VF in all except for one patient who remained on amiodarone
Brugada J et al (20)	Case series	14	All have preexisting ICD	Ablation of the substrate identified in the presence of flecainide can eliminate the BrS phenotype
Sacher et al (26)	Case report	1	Not reported	Ajmaline helps with identifying substrate during epicardial mapping
Széplaki G et al (27)	Case report	1	No pre-existing ICD	Case report of patient who has successful epicardial ablation ICD not implanted
Maeda S et al (28)	Case report	1	Pre-existing ICD	Bipolar epicardial mapping and ablation in a patient with Brugada and coexistent J wave
Chung FP et al. (22)	Case series	15	Not reported	Epicardial warm water instillation enhances substrates and the increases VT/VF inducibility
Pappone C et al (29)	Prospective study	135	All have preexisting ICD	Ajmaline-assisted substrate-based ablation is effective
Fernandes GC (30)	Systematic review	233	N/A	Epicardial ablation is more effective than endocardial ablation in preventing VT/VF
BRAVO Trial (31)	Registry	159	All have preexisting ICD except for five	Ablation treatment is safe and highly effective in preventing VF recurrence in high-risk BrS

**Fig. 3:**
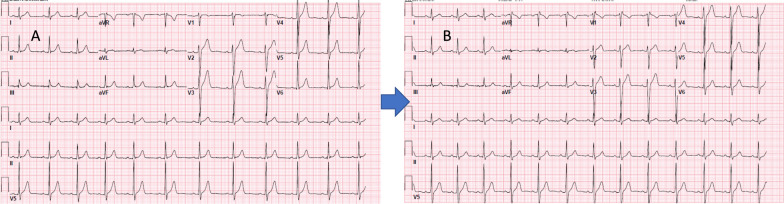
**A** baseline ECG showing type 3 Brugada ECG pattern in lead V1 **B** ECG after the administration of flecainide showing coved ST segment elevation

**Fig. 4:**
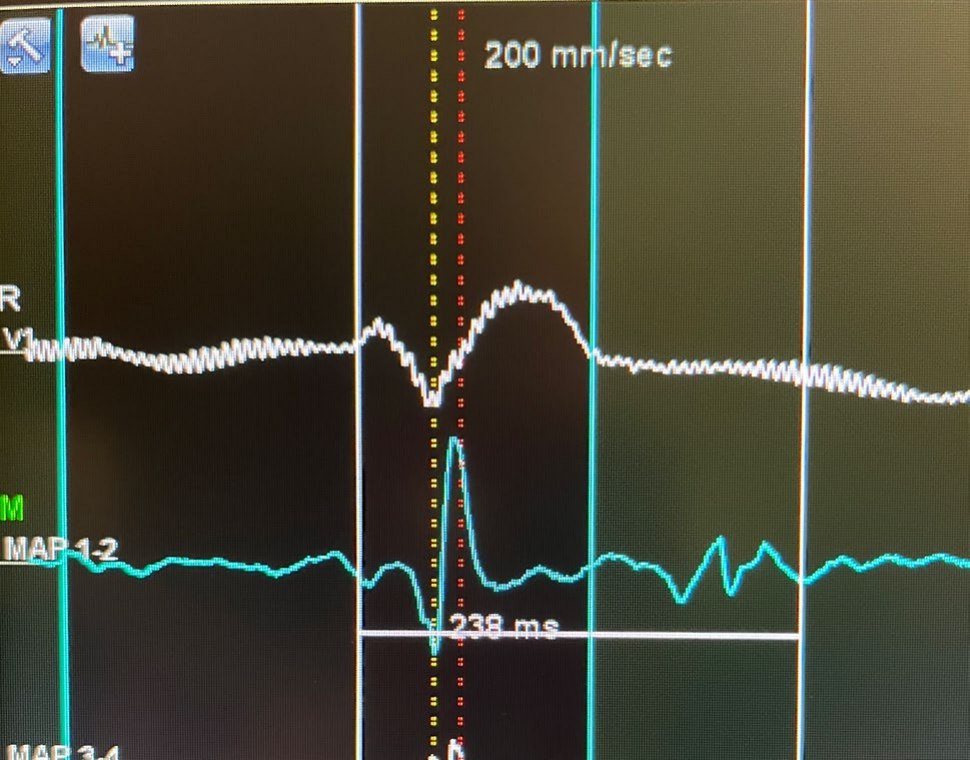
.

While sudden death can be the initial manifestation of BrS, the majority of patients typically present with a history of recurrent syncope, particularly during rest. Several risk factors have been associated with an increased likelihood of arrhythmia in individuals with BrS. These include the use of medications that block sodium current (INa) channels and tricyclic antidepressants, as well as fever due to the temperature sensitivity of the sodium voltage-gated channel alpha subunit 5 (SCN5A) channel (Figs. 3 and 4).

Additionally, the consumption of heavy meals has been identified as a potential risk factor for arrhythmia development in BrS syndrome.

### Genetics

In the context of BrS syndrome, the SCN5A gene is the most commonly implicated gene, often associated with loss-of-function mutations. This is in contrast to long QT syndrome type 3, where SCN5A mutations result in gain-of-function. It has been reported that nearly 25% of individuals with BrS carry variants in the SCN5A gene. Given the genetic basis of the syndrome, clinical and genetic testing of probands (the first identified affected individuals) is strongly recommended not only for diagnostic purposes but also for the purpose of family screening [4].

In the case of BrS, it is important to extend clinical and genetic testing to all first- and second-degree relatives, even if they do not exhibit the characteristic phenotype associated with the syndrome. This comprehensive testing approach is crucial in providing accurate genetic counseling and identifying individuals who may be at risk of developing the syndrome or passing it on to future generations.

Clinicians managing patients with BrS should adopt an aggressive approach in avoiding and treating fever. Fever has been recognized as a trigger for arrhythmias in individuals with BrS due to the temperature sensitivity of the SCN5A channel. Therefore, prompt management of fever is essential to reduce the risk of arrhythmic events.

Furthermore, it is important for clinicians to be cautious with the use of medications that block the SCN5A channel in patients with BrS. To aid healthcare professionals in making informed decisions regarding drug safety, resources such as www.brugadadrugs.org [5] provide valuable information on drugs to be avoided in individuals with BrS, considering their potential to block the SCN5A channel and increase the risk of arrhythmias.

By understanding the genetic basis of BrS, conducting thorough clinical and genetic testing, and implementing appropriate management strategies, healthcare professionals can effectively counsel individuals and families affected by the syndrome, mitigate the risk of adverse events, and enhance patient care.

### Risk stratification

Stratifying the risk of sudden cardiac death (SCD) in patients with BrS is a challenging yet essential task, considering that the first manifestation of the syndrome for most patients is cardiac arrest or sudden cardiac death. Consequently, the identification and management of asymptomatic individuals at high risk of sudden death pose significant challenges within the realm of BrS management [6].

In patients who have experienced cardiac arrest or are presenting with presumed arrhythmic syncope, risk stratification strategies may have limited utility since these individuals are already recognized to be at a heightened risk of adverse cardiac events. However, for asymptomatic patients, risk stratification becomes of paramount importance to guide appropriate management decisions and preventive interventions.

Currently, the guidelines neither explicitly endorse nor discourage the use of electrophysiological studies and ventricular tachycardia/ventricular fibrillation (VT/VF) inducibility patterns for risk stratification in patients with BrS [1]. The role of these methods in predicting future arrhythmic events and guiding therapeutic interventions remains to be further elucidated.

In addition to invasive techniques, several noninvasive risk stratification markers have been proposed, with signal-averaged electrocardiograms (ECGs) being one such example [7]. However, it is important to note that the available evidence supporting these markers is derived primarily from small observational studies. Consequently, further validation in larger series is necessary to establish their utility and reliability in risk stratification for BrS.

Given the potentially catastrophic consequences associated with SCD in BrS, efforts to refine risk stratification strategies and identify novel predictive markers are ongoing. Future research endeavors aimed at establishing standardized risk stratification approaches and incorporating both invasive and noninvasive methods hold promise in improving the management and outcomes of individuals with BrS.

### Management

The management of patients with BrS poses significant challenges due to the complex nature of the condition. Implantable cardioverter–defibrillators (ICDs) play a crucial role in preventing sudden cardiac death and are recommended for specific patient groups. According to the guidelines, ICD implantation is considered a Class I indication for patients with BrS and a history of prior cardiac arrest. Furthermore, individuals with BrS who exhibit a spontaneous type I electrocardiographic pattern and experience recurrent syncope are also candidates for ICD placement [8].

While ICDs effectively reduce the risk of sudden cardiac death, it is important to acknowledge the potential risks and complications associated with these devices throughout the patient's lifetime. This is particularly relevant for younger patients who undergo device implantation, as they may face long-term challenges related to device performance, battery life, and the need for device replacement [9].

In addition to ICD therapy, comprehensive management of BrS involves patient education and awareness. It is strongly recommended by the European Society of Cardiology (ESC) guidelines that all individuals diagnosed with BrS be educated about the factors that can modulate or precipitate arrhythmias and advised on how to avoid these triggers [10]. By providing patients with appropriate knowledge and guidance, the aim is to empower them to actively participate in their own care and minimize the risk of potentially life-threatening events.

In terms of pharmacological treatment, quinidine is currently the only known effective long-term therapy for BrS. It has demonstrated benefits in reducing arrhythmic events and is considered a viable option for patients who require medical intervention [11]. However, the selection of medication should be individualized based on the patient's specific clinical characteristics and underlying risk profile.

For asymptomatic patients with BrS who do not exhibit a spontaneous type I electrocardiographic pattern, the risk of arrhythmic events is generally low. In such cases, the implantation of an ICD is not recommended, even if a sodium channel-blocking drug evokes a type I pattern on the electrocardiogram [12]. Close monitoring and regular follow-up are important for these individuals to detect any changes in their clinical status and promptly initiate appropriate interventions if needed.

The management of BrS requires a multidisciplinary approach involving cardiologists, electrophysiologists, and other healthcare professionals to ensure optimal patient care and minimize the risk of adverse outcomes. Ongoing research and advancements in both pharmacological and non-pharmacological therapies are essential for further improving the management strategies and outcomes for individuals with BrS.

### Epicardial ablation in Brugada syndrome

The standard therapy for the prevention of SCD in BrS is an implantable cardioverter–defibrillator (ICD) in patients who have experienced a prior cardiac arrest or syncopal events [13]. However, an ICD does not prevent the occurrence of VF but only terminates malignant ventricular arrhythmias. Anti-arrhythmic medications such as quinidine can be used to prevent malignant ventricular arrhythmias in BrS patients, but such medications are not well tolerated and are associated with side effects [11] such as thrombocytopenia. Therefore, the need for an alternative therapy has led to attempts in using catheter ablation to treat the arrhythmogenic substrate of BrS.

The treatment of BrS patients with recurrent VF using catheter ablation was first introduced by Haïssaguerre et al. [14]. The approach involved endocardial ablation of focal triggers of malignant ventricular arrhythmias originating predominantly from the right ventricular outflow tract (RVOT). However, this approach is limited by mapping premature ventricular complexes (VF triggers) during the procedure which can be challenging since premature ventricular beats rarely occur (< 1 beat per hour) in BrS based on Holter monitoring findings [15]. Subsequently, a BrS animal model study demonstrated that epicardial ablation on the right ventricle was successful in eliminating ventricular arrhythmia compared to endocardial approach [16]. This concept was later successfully applied in humans when Nademanee et al. observed that RVOT epicardial ablation of VF substrate in BrS patients with frequent electrical storms proved to be effective in controlling ventricular arrhythmias during follow-up in eight of the nine patients [17]. All nine patients had abnormal intracardiac electrocardiograms characterized by low voltage (< 1mV), prolonged duration (> 80 ms), and fractionated late potentials on the anterior RVOT epicardium. Ablation at these sites successfully rendered malignant arrhythmia non-inducible with no recurrence of VT or VF in all except for one patient who remained on amiodarone. Interestingly, in BrS VF victims it was noted that there is increased fibrosis and collagen content in the RVOT epicardium [18] that may explain the abnormal and fractionated signals despite the absence of structural changes based on cardiac MRI findings.

The use of abnormal intracardiac electrocardiogram, also called arrhythmic electrophysiological substrate (ASE) [19], described above as a well-defined potentially reversible arrhythmogenic substrate on the anterior epicardial RVOT with an endpoint of non-inducibility of VT/VF has changed the landscape of managing BrS. Furthermore, it has been shown that with the use of Ajmaline (administered as 1mg/kg over 5 min) in BrS patients, abnormally prolonged and fragmented epicardial potentials are revealed and can be used as targets for catheter ablation [1]. If Ajmaline is not available, other class I agents can also be used such as flecainide (2 mg/kg per 10 min) [20] or procainamide. The ablation of ASE has been demonstrated in multiple studies to reduce recurrence of malignant arrhythmia and interestingly it has been shown to normalize ECG. In fact, the main predictor of VF recurrence necessitating repeat ablation is the continuing presence of Brugada ECG pattern [21].

The use of catheter ablation has a very promising role in managing BrS patients; however, further studies are needed to assert its long-term benefit in preventing recurrent VF. Currently, the randomized controlled trial BRAVE is investigating the benefit of catheter ablation as a potential curative treatment strategy in patients with BrS. Meanwhile, catheter ablation can be used in patients with recurrent VT/VF in experienced centers and patients should be counseled about the risks associated with epicardial access and the potential arrhythmogenic effects of drug administration.

## Conclusions

Brugada syndrome, an inherited condition characterized by ventricular arrhythmia leading to sudden cardiac death, has undergone significant advancements in its management since its initial recognition. Currently, the implantation of an implantable cardioverter–defibrillator (ICD) is indicated for patients who have experienced sudden cardiac arrest, documented ventricular arrhythmia, and/or syncope. Although quinidine can be employed to manage recurrent ventricular arrhythmia, it is associated with notable side effects. A significant milestone in the management of BrS patients with recurrent ventricular arrhythmia has been the introduction of catheter ablation, particularly the epicardial approach. However, further studies are required to establish the efficacy of this intervention.

## Data Availability

Not applicable.

## Abbreviations

BrS: Brugada syndrome
ECG: Electrocardiogram
VT: Ventricular tachycardia
VF: Ventricular fibrillation
ICD: Implanted cardioverter–defibrillator
EPS: Electrophysiological stimulation
SCD: Sudden cardiac death
SD: Sudden death
INa: Sodium current
SCN5A: Sodium voltage-gated channel alpha subunit 5
ICS: Intercostal space
